# Human tendon adaptation in response to mechanical loading: a systematic review and meta-analysis of exercise intervention studies on healthy adults

**DOI:** 10.1186/s40798-015-0009-9

**Published:** 2015-03-27

**Authors:** Sebastian Bohm, Falk Mersmann, Adamantios Arampatzis

**Affiliations:** Department of Training and Movement Sciences, Humboldt-Universität zu Berlin, Philippstr. 13, Haus 11, 10115 Berlin, Germany

## Abstract

**Background:**

The present article systematically reviews recent literature on the *in vivo* adaptation of asymptomatic human tendons following increased chronic mechanical loading, and meta-analyzes the loading conditions, intervention outcomes, as well as methodological aspects.

**Methods:**

The search was performed in the databases PubMed, Web of Knowledge, and Scopus as well as in the reference lists of the eligible articles. A study was included if it conducted (a) a longitudinal exercise intervention (≥8 weeks) on (b) healthy humans (18 to 50 years), (c) investigating the effects on mechanical (i.e., stiffness), material (i.e., Young’s modulus) and/or morphological properties (i.e., cross-sectional area (CSA)) of tendons *in vivo*, and was reported (d) in English language. Weighted average effect sizes (SMD, random-effects) and heterogeneity (Q and *I*^*2*^ statistics) of the intervention-induced changes of tendon stiffness, Young’s modulus, and CSA were calculated. A subgroup analysis was conducted regarding the applied loading intensity, muscle contraction type, and intervention duration. Further, the methodological study quality and the risk of bias were assessed.

**Results:**

The review process yielded 27 studies with 37 separate interventions on either the Achilles or patellar tendon (264 participants). SMD was 0.70 (confidence interval: 0.51, 0.88) for tendon stiffness (*N*=37), 0.69 (0.36, 1.03) for Young’s modulus (*N*=17), and 0.24 (0.07, 0.42) for CSA (*N*=33), with significant overall intervention effects (*p*<0.05). The heterogeneity analysis (stiffness: *I*^*2*^=30%; Young’s modulus: *I*^*2*^=57%; CSA: *I*^*2*^=21%) indicated that differences in the loading conditions may affect the adaptive responses. The subgroup analysis confirmed that stiffness adaptation significantly (*p*<0.05) depends on loading intensity (*I*^*2*^=0%), but not on muscle contraction type. Although not significantly different, SMD was higher for interventions with longer duration (≥12 weeks). The average score of 71±9% in methodological quality assessment indicated an appropriate quality of most studies.

**Conclusions:**

The present meta-analysis provides elaborate statistical evidence that tendons are highly responsive to diverse loading regimens. However, the data strongly suggests that loading magnitude in particular plays a key role for tendon adaptation in contrast to muscle contraction type. Furthermore, intervention-induced changes in tendon stiffness seem to be more attributed to adaptations of the material rather than morphological properties.

## Key points

- Tendons are highly responsive to increased mechanical loading and adapt through changes of their mechanical, material, and morphological properties.
- Changes in tendon stiffness seem to be more attributed to adaptations of the material rather than morphological properties.
- An effective training intervention for the tendon should apply a high loading intensity over a longer intervention duration (>12 weeks).

## Background

Tendons transmit the force exerted by the corresponding muscle to the skeleton and, therefore, are crucial components for human locomotion [1-3]. Further, the non-rigidity of tendons allows the storage and return of strain energy during locomotion [4,5] and facilitates the muscle force potential due to the force-length-velocity relationship [6-8]. Hence, tendon properties not only affect human daily locomotion like walking/running [9] and stability performance [10], but also significantly determine athletic performances, e.g., sprinting [11,12] and jumping [8,13,14] as well as the economy of running [15-17]. Furthermore, tendons are sensitive to their mechanical environment [18-22]. Following a period of enhanced mechanical loading, tendon stiffness may increase [23-26] to maintain physiological ranges of strain during locomotion, since the ultimate tendon strain is more or less constant [27]. Two mechanisms could account for an increase of tendon stiffness: a) changes of the tendon material (i.e., increase of Young’s modulus) and b) changes of the tendon morphological properties (i.e., increase of cross-sectional area) [24,28-31]. Both tendon material and morphological changes result not only from an increase of collagen synthesis but also from changes of collagen fibril morphology and levels of collagen molecular cross-linking [19,32,33]. Besides physiological adaptive responses, excessive mechanical loading (i.e., overloading) was considered as an important factor in the etiology of tendinopathy [20,34,35], which is associated with pain, focal tendon tenderness, and decreased strength and movement [32].

The development and improvement of measurement techniques in the past 15 years, especially the measurement of tendon elongation during muscle contractions by means of an ultrasound-based methodology as well as the determination of the tendon cross-sectional area (CSA) from magnetic resonance images (MRI), enabled researchers to investigate human tendon mechanical, material, and morphological properties *in vivo* and adaptive responses following chronic increased loading [2,19,36]. Kubo et al. [37] were the first who reported an increase in stiffness and Young’s modulus of the patellar tendon in humans following 12 weeks of exercise-based loading. An intervention-induced region specific hypertrophy of the patellar and Achilles tendon were initially reported in 2007 by Kongsgaard et al. [24] and Arampatzis et al. [29], respectively. To date, a lot of experimental studies evidenced the adaptive potential of tendons following exercise interventions, which featured different levels of mechanical loading conditions (e.g., intensity, duration of a single loading cycle, repetitions, sets, intervention duration, and training frequency per week) [25,28,30,31,38-41]. Since some interventions reported greater adaptive tendon responses than others, the outcome of the studies seems to be affected by differences of the applied loading conditions. This means that the levels of the loading conditions may determine the material and morphological adaptive responses of tendons. Although some studies investigated the effect of different loading levels (i.e., load magnitude [24,29], loading rate [31], and load duration [31,37,42]) on tendon adaptation, the small sample sizes of 8 to 14 participants used in these studies limit the generalizability of the outcomes. A meta-analysis of relevant experimental studies that examines the interaction of the levels of loading conditions with respect to study outcome could deepen our understanding of the effectiveness of certain loading levels on tendon adaptation. Furthermore, different methodological approaches could have affected the study outcomes, thus, additionally challenging the generalization of the findings. For example, most of recent studies on tendon adaptation used a manual segmentation of magnetic resonance or ultrasonographic images to determine the tendon CSA. However, using ultrasound images instead of MRI for the manual segmentation [38,39,43,44], intervention-induced changes of the tendon CSA might have been undetected or overrated, since the reliability of this manual segmentation method was reported to be poor [45]. Considering the methodological quality (i.e., internal, statistical, external validity aspects) of each study in a systematic meta-analysis would further improve our knowledge regarding mechanical loading and tendon adaptation.

Therefore, the objectives of the present study are to systematically review recent literature reports (i.e., longitudinal study designs) on the adaptation of asymptomatic human tendons following increased mechanical loading (i.e., training intervention) *in vivo* and to meta-analyze the applied levels of loading conditions, intervention outcomes, as well as methodological aspects, which has yet to be conducted. For a complete description of the adaptive processes, we will consider tendon mechanical, material, and morphological properties. Particular attention is given to the effect of loading intensity, muscle contraction type, and intervention duration on tendon adaptive responses by performing a respective subgroup analysis. This meta-analysis may provide crucial information on how to facilitate tendon adaptation.

## Methods

### Search strategy

The search was performed by using the electronic bibliographic databases ISI Web of Knowledge, PubMed, and Scopus (1970 to November 2014) and by screening the reference lists of the eligible articles. The following keyword combinations (i.e., search operator AND) were separately applied in the database search (i.e., title, abstract, keywords): tendon properties adaptation, tendon stiffness adaptation, tendon function adaptation, tendon mechanical loading adaptation, tendon properties training, and tendon properties exercise.

### Study selection and inclusion criteria

**Figure 1:**
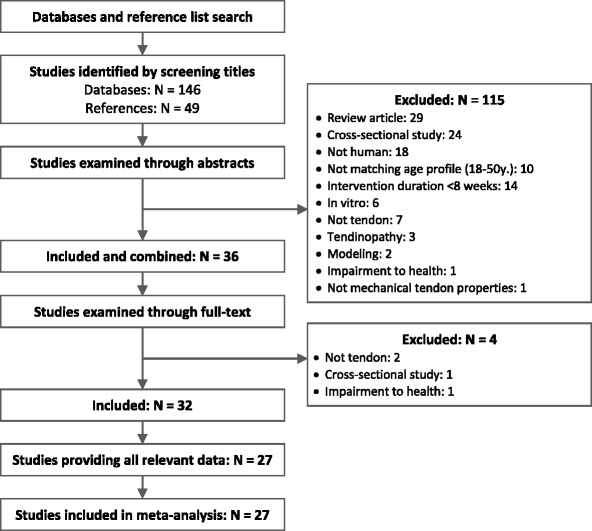
**Flowchart of the systematic review process.**

Two independent reviewers (S.B. and F.M.) evaluated the titles of the studies that resulted from the search and included studies when the title indicated that the following inclusion criteria were fulfilled: (a) a longitudinal exercise intervention (≥8 weeks) was conducted, (b) healthy humans (18 to 50 years) served as participants, and (c) the effects on mechanical (stiffness), material (Young’s modulus), and/or morphological (CSA) properties of asymptomatic tendons *in vivo* were reported (d) in the English language. The abstracts and, thereafter, the full text of the identified studies were then examined to confirm the inclusion. If a study did not meet all criteria, the respective exclusion criterion was documented and the study was eliminated from further analysis. In the case of disagreement of the two reviewers, a third reviewer (A.A.) was consulted. Figure 1 illustrates the systematic review process of the present meta-analysis.

### Methodological quality and risk of bias

A customized methodological quality scale was designed to assess the internal, statistical, and external validity of the included studies in regard to the conceptual definition (Table 1). A positive point was assigned when a specific quality criterion was fulfilled (Table 1). However, if a criterion could not be scored because it was not part of the study (e.g., mechanical but not morphological tendon properties were investigated), the criterion was excluded from the further quality assessment of the study. The quality score of each validity aspect (i.e., internal, statistical, and external) was calculated by dividing the number of items with a positive score by the total number of items (the quotient was then multiplied by 100). The scores were averaged to calculate the overall methodological quality of each study. The assessment of the risk of bias (sequence generation, allocation concealment, blinding outcome assessor, incomplete outcome data, selective outcome reporting, other sources of bias) was based on the Cochrane Risk of Bias tool [46]. The data extraction and scoring were performed by two independent observers (S.B. and F.M.), and in the case of disagreement, a third one was consulted (A.A.). A funnel plot of the tendon stiffness from all included studies was created to estimate publication bias.

**Table 1:** **Criteria of the methodological quality**

	**Scoring**
**Internal validity**	
1. Study design	A positive point was assigned if the following aspects were considered:
	1. Mechanical tendon properties (stiffness)
	2. Material tendon properties (Young’s modulus)
	3. Morphological tendon properties (cross-sectional area)
	4. Control group (no specific training) was included and participants were randomly assigned
2. Methods	A positive point was assigned if the following aspects were considered:
2.1 Mechanical properties	
• Object of investigation	A. Only the free tendon was assessed [79]
• Calculation of tendon force	B. Consideration of gravitational forces [80,81]
	C. Consideration of axes misalignment of dynamometer and joint [80-82]
	D. Consideration of antagonistic muscle activation [83,84]
	E. Tendon lever arm directly measured for each subject
• Measurement of tendon elongation	F. Consideration of joint angle changes during the maximal isometric contraction on the tendon elongation measurement [84,85]
	G. Using the average of multiple trials (>1) to increase the reliability of the ultrasound technique [86]
2.2 Morphological properties	A. Magnetic resonance imaging was used [45,76]
	B. Different positions along tendon length were assessed to account for potential region specific adaptations [24,29,30]
3. Cofactors	A positive point was assigned if the following aspects were considered:
	A. Influence of gender
	B. Influence of physical activity level of the participants
**Statistical validity**	
4. Statistical tests	A positive point was assigned if appropriate statistical tests were used
5. Power analysis	A positive point was assigned if effect sizes were calculated and reported
**External validity**	
6. Eligibility of sample and variable	A positive point was assigned if the intervention included:
	1. Appropriate participant sample
	2. Appropriate variables
7. Description of the exercise intervention protocol	A positive point was assigned if the following criteria were reported:
	A. Intensity of muscle contraction
	B. Duration of single stimulus
	C. Repetitions per set
	D. Number of sets
	E. Number of weeks of intervention
	F. Number of trainings per week
8. Description of the participant sample	A positive point was assigned if the following criteria were reported:
	A Age, B Gender, C Body height, D Body weight, E Activity level

### Data extraction

One reviewer (S.B.) extracted the following data from the full-text articles of all included studies, and a second reviewer (F.M.) confirmed the extraction. The data were merged in a table, including the information of the source (name of the first author and year of publication), the label of the participant sample in the respective study (i.e., experimental or control group according to the inclusion criteria), the characteristics of the participants (i.e., number, gender, activity level, investigated tendon), the performed intervention with the respective loading conditions (i.e., type of training, intensity, duration of single loading cycle, repetitions, sets, intervention duration, and training frequency), and the outcome of the study for either tendon stiffness, Young’s modulus, and CSA as the difference of pre and post values in percentage (i.e., ((post value − pre value)/pre value) * 100) with the corresponding significance indication. Furthermore, the part of the tendon (i.e., free tendon or tendon-aponeurosis complex) that was used for the parameter calculation was documented. In studies where both calculation approaches were used, only the values from the free tendon were included. If the stiffness or Young’s modulus was calculated within several percentage intervals of the force-elongation or stress-strain relationship, the values from the highest interval were used. In case that the CSA was reported for different positions along the tendon length, the mean value and pooled standard deviation were calculated and included. If the required data (i.e., means and standard deviations of pre- and post-intervention values) were not reported in the article or presented in an inappropriate format for data extraction (e.g., graph instead of values), the corresponding authors were contacted and asked to provide the missing values. Extracting values visually from a graph was the final option. In case the relevant data were not available, the study was excluded.

### Statistical analysis

In order to assess the impact of mechanical loading on tendon adaptation, the effect sizes of the intervention-induced changes (i.e., changes to baseline) of the tendon stiffness, Young’s modulus, and CSA for each study were calculated. As the stiffness, Young’s modulus, and CSA were not always measured using identical methodological approaches, the effect size was calculated as the standardized mean difference (SMD) [47]. The SMD included further an adjustment (Hedges’ adjusted *g*) for small sample bias [47]. Throughout the following manuscript, we will use the term SMD when referring to effect sizes of individual studies. The SMDs from all studies were then pooled in a meta-analysis to estimate the weighted average effect size of the tendon stiffness, Young’s modulus, and CSA [47,48]. Thereto, we used a random-effects model of the generic inverse variance method, which gives more weight to larger studies (i.e., smaller standard errors) and accounts for heterogeneity of the included studies [47,49]. To analyze the presence of an overall intervention effect on the tendon stiffness, Young’s modulus, and CSA, a test statistic (i.e., null hypothesis: no overall effect of the intervention) was performed [47]. A forest plot was created to illustrate the SMDs and 95% confidence intervals (CIs) of tendon stiffness, Young’s modulus, and CSA for all respective studies as well as the overall effect. Further, heterogeneity between study outcomes was investigated using *Q* and *I*^2^ statistics to assess if differences between outcomes are due to study diversity rather than chance [50]. A subgroup analysis was conducted on the following loading conditions: intensity (i.e., higher versus lower than 70% of maximum voluntary contraction (MVC) or one repetition maximum (RM)), muscle contraction type (i.e., isometric, eccentric, concentric-eccentric), and intervention duration (i.e., shorter and longer than 12 weeks). A second forest plot was designed to present the SMDs and CI of tendon stiffness between studies, which applied low and high loading intensities, respectively. Statistical procedures were performed by means of the software Review Manager v.5.2 [51].

## Results

### Literature search

The search by the defined keywords yielded 3,944 hits in the three databases. After screening all study titles and eliminating duplicates from the different databases, 146 potentially eligible studies were identified. Following the abstract examination, 35 studies remained included; however, the full text assessment showed that four more studies did not confirm all criteria and, thus, were excluded from the further analysis. The screening of the reference lists of the included studies provided a number of 49 potentially eligible studies. However, except one study, all articles did not meet the criteria or were already included. Five studies were excluded from the remaining 32 due to a lack of relevant information about the loading conditions [52,53] or outcome values [44,54,55]. Finally, 27 studies fulfilled all criteria and were included in the present meta-analysis (Figure 1).

### Description of the included studies

All included studies assessed the effect of mechanical loading on either the patellar tendon (*N* = 12) or the Achilles tendon (*N* = 15). Nine studies applied a different loading protocol on the two legs of the participants of the exercise group, and one study investigated three different intervention groups. In the present meta-analysis, each of these interventions was treated as a separate intervention. When a study presented the data of different intervention groups, but not all of them fulfilled the inclusion criteria, only the ones that met all criteria were included. An overweighting of single studies within the meta-analysis (i.e., bias) due to this approach was, however, not expected, as the loading conditions between the separate interventions were different and independent. In fact, only this procedure allowed to include all available data for a representative meta-analysis. The articles from Foure et al. [38,56,57] reported the effect of a single intervention on different parameters of the Achilles tendon, as indicated by the same number of participants with similar anthropometrics, identical training protocol, and values of tendon CSA. The relevant parameters for the present analysis (i.e., tendon stiffness and CSA) were extracted and considered as a single intervention. Furthermore, Kubo et al. [37,42] presented the data of one intervention in two articles, as indicated by the same number of participants and anthropometrics, training protocol, and results of tendon stiffness and CSA (LC protocol exercise group in [42]). Thus, the values were only included once. In the study of Kubo et al. [58], the authors compared the results of two previous investigations [59,60] that were already included in the present meta-analysis under a new research question. These results were also not considered as a new investigation. In another article [24], the CSA pre- and post-intervention values were exclusively reported in a graph (figure four, page 116). The respective means and standard error of means were visually extracted from the graph and used in order to calculate the standard deviation and SMD.

Finally, the present meta-analysis included in total 37 interventions (participants in total *N* = 264) eligible for the research question, and their characteristics are summarized in Table 2. In all 37 interventions, the parameter tendon stiffness was used in order to quantify the training effect on the adaptive tendon responses. Thirty-three of these also examined the tendon CSA, and 17 studies further included the parameter Young’s modulus. Seventeen interventions applied the mechanical stimulus on the tendon by means of isometric muscle contractions, 11 interventions used a combination of concentric and eccentric contractions or solely concentric (*N* = 1) or eccentric contractions (*N* = 3), 5 interventions performed plyometric training, 1 intervention added stretching to the resistance training, and 1 study investigated the effect of running on the tendon properties (Table 2). The loading conditions were set to different levels between studies, using high and low intensities, short and long durations of the single loading, and different numbers of repetitions and sets (Table 2). However, only three studies (i.e., eight interventions) specified the corresponding tendon strain magnitude to the muscle contraction intensity [28,29,31]. Thirty-five of the 37 interventions were performed for 8 to 14 weeks, and the participants exercised on 2 to 4 days per week. Except for four interventions [29,61,62], which included both female and male participants and one intervention including solely women [63], all other interventions were performed with men. In almost all studies, the participants were regularly physically active, but not involved in intensive sports activity. One intervention was performed with cricket players [39] and another one with runners [17]. The number of exercised participants ranged between studies from 6 to 15 with a mean of 9.8 ± 2.3.

**Table 2:** **Data extraction from the included studies**

**Source**			**Participants**			**Intervention**								**Outcome**							
**Reference**	**Year**	**Group**	***N***	**Sex**	**Activity level**	**Tendon**	**Type of training**	**Intensity**	**Duration**	**Reps**	**Sets**	**Weeks**	**Times/week**	**Stiffness**			**YM**		**CSA**		
														**Location**	**%**	**Sig**	**%**	**Sig**	**Location**	**%**	**Sig**
Albracht et al. [15]	2013	EP	13	m	Run	AT	Is (rep)	90% MVC	3 s	4	5	14	4	Ap (GM F)	15.8	*					
Arampatzis et al. [29]	2010	EP	11	m	Reg	AT	Is (rep)	55% MVC	1 s	20	5	14	4	Ap (GM F)	−5.2	-	−4.8	-	Free	1.3	-
		EP	11	m	Reg	AT	Is (rep)	90% MVC	1 s	12	5	14	4	Ap (GM F)	17.1	*	16.9	*	Free	0.5	-
Arampatzis et al. [28]	2007	EP	11	f, m	Reg	AT	Is (rep)	55% MVC	3 s	7	5	14	4	Ap (GM F)	7.9	-	−1.6	-	Free	4.3	-
		EP	11	f, m	Reg	AT	Is (rep)	90% MVC	3 s	4	5	14	4	Ap (GM F)	36.0	*	22.9	*	Free	9.6	*
Bohm et al. [31]	2014	EP	12	m	Reg	AT	Is (sta)	90% MVC	12 s	1	5	14	4	Ap (GM M)	24.8	*	17.7	*	Free	5.3	*
		EP	12	m	Reg	AT	Is (rep)	90% MVC	3 s	4	5	14	4	Ap (GM M)	53.9	*	45.2	*	Free	4.4	*
		EP	14	m	Reg	AT	Ply	90% MVC	Approximately 0.26 s	72	5	14	4	Ap (GM M)	28.4	-	19.6	-	Free	2.5	-
		EP	14	m	Reg	AT	Is (rep)	90% MVC	3 s	4	5	14	4	Ap (GM M)	37.3	*	36.3	*	Free	3.7	*
Carroll et al. [61]	2011	CG	7 (11)	f, m	Unt	PT	Co-Ec (rep)	74% RM	nr	2 to 3	5 to 10	12	3	Free	13.9	+	18.4	*	Free	−1.7	-
Fletcher et al. [17]	2010	EP	6	m	Run	AT	Is (sta)	80% MVC	20 s	1	4	8	3	Ap (GM F)	18.6	-					
Fouré et al. [71]	2009	EP	6	m	Exp	AT	Ply	nr	nr	150 to 280	nr	8	2	Ap (GM M)	4.1	-					
Foure et al. [38,56,57]	2010a,b,2011	EP	9	m	Reg	AT	Ply	nr	nr	200 to 600	nr	14	2.4	Ap (GM M)	26.5	*			Free	3.1	-
Foure et al. [43]	2013	EP	11	m	Reg	AT	Ec (rep)	nr	nr	200 to 600	nr	14	2.4	Ap (GM F)	16.4	-			Free	−1.5	-
Hansen et al. [62]	2003	EP	11	f, m	Unt	AT	Run	nr	30 to 50 min	1		34	2.4	Ap (GM F)	7.3	-			Free	−0.3	-
Houghton et al. [39]	2013	EP	7	nr	Cri	AT	Ply	nr	nr	4 to 10	2 to 6	8	1.9	Ap (GM M)	−8.9	-	−20	-	Free	12.9	*
Kongsgaard et al. [24]	2007	EP	12	m	Unt	PT	Co-Ec (rep)	70% RM	nr	8	10	12	3	Free	14.6	*	12.2	-	Free	3.3	nr
		EP	12	m	Unt	PT	Co-Ec (rep)	16% RM	nr	36	10	12	3	Free	−9.2	-	−4.2	-	Free	1.5	nr
Kubo et al. [37,42]	2001a	EP	8	m	Reg	PT	Is (rep)	70% MVC	Rapid	50	3	12	4	Ap (VL F)	17.5	-			Free	1.4	-
	2001a,b	EP	8	m	Reg	PT	Is (sta)	70% MVC	20 s	1	4	12	4	Ap (VL F)	57.3	*	50.3	*	Free	1.4	-
Kubo et al. [25]	2002	EP	8	m	Reg	AT	Co-Ec (rep)	70% RM	nr	10	5	8	4	Ap (GM F)	31.3	*			Free	−3.3	-
		EP	8	m	Reg	AT	Co-Ec (rep) + S	70% RM	nr + 45 s	10 + 5	5 + 1	8	4 + 7 (2×/day)	Ap (GM F)	23.8	*			Free	3.4	-
Kubo et al. [63]	2003	EP2	11	f	Reg	PT	Co-Ec (rep)	BW	nr	44	1	24	6	Ap (VL F)	15.7	-					
Kubo et al. [40]	2006a	EP	9	m	nr	PT	Is (sta) [50°]	70% MVC	15 s	1	6	12	4	Ap (VL F)	9.7	-			Free	1.5	-
		EP	9	m	nr	PT	Is (sta) [100°]	70% MVC	15 s	1	6	12	4	Ap (VL F)	50.9	*			Free	1.5	-
Kubo et al. [87]	2006b	EP	8	m	Reg	PT	Is (sta)	70% MVC	15 s	1	10	12	4	Free	−0.2	-			Free	0.3	-
Kubo et al. [88]	2006c	CG	9	m	nr	PT	Co-Ec (rep)	80% RM	4 s	10	4	12	3	Free	8.5	-			Free	−0.6	-
Kubo et al. [59]	2007	EP	10	m	Unt	AT	Ply	40% RM	nr	10	5	12	4	Ap (GM M)	19.4	-			Free	3.3	-
		EP	10	m	Unt	AT	Co-Ec (rep)	80% RM	4 s	10	5	12	4	Ap (GM M)	29.7	*			Free	−1.2	-
Kubo et al. [60]	2009	EP	10	m	nr	PT	Is (sta)	70% MVC	15 s	1	10	12	4	Free	71.1	*			Free	4.0	-
		EP	10	m	nr	PT	Co-Ec (rep)	80% RM	4 s	10	5	12	4	Free	25.4	-			Free	1.3	-
Kubo et al. [52]	2010	EP	8	m	Reg	PT	Is (sta)	70% MVC	15 s	1	10	12	4	Ap (VL F)	50.9	*			Free	1.0	-
Kubo et al. [72]	2012	EP	9	m	Reg	AT	Is (sta)	80% MVC	15 s	1	15	12	4	Ap (GM M)	51.4	*			Free	2.7	-
Malliaras et al. [41]	2013	EP	9	m	Reg	PT	Co (rep)	80% RM	5 s	7 to 8	4	12	3	Free	49.9	-	52	-	Free	5.0	-
		EP	10	m	Reg	PT	Ec (rep)	80% RM	5 s	12 to 15	4	12	3	Free	39.2	-	38.6	-	Free	3.6	-
		EP	10	m	Reg	PT	Ec (rep)	80% RM (Ec)	5 s	7 to 8	4	12	3	Free	80.9	*	77.3	*	Free	5.8	-
Seynnes et al. [30]	2009	EP	15	m	Reg	PT	Co-Ec (rep)	80% RM	nr	10	4	9	3	Free	22.7	*	18.4	*	Free	3.9	*

Group (i.e., as assigned in the respective article): EP, experimental group; CG, control group. Sex: f, female; m, male. Activity level: Reg, regularly physically active and recreational sports; Unt, untrained; Exp, explosive sports (i.e., volleyball, basketball, handball); Run, runners; Cri, cricket players. Tendon: PT, patellar tendon; AT, Achilles tendon. Type of training: Is, isometric muscle contraction; Co, concentric; Ec, eccentric; Ply, plyometric; Run, running; S, stretching; rep, repetitive; sta, static. Intensity: MVC, maximum voluntary contraction; RM, one repetition maximum; BW, body weight. Outcome: YM, tendon Young’s modulus; CSA, tendon cross-sectional area. Location (i.e., refers to the anatomical structure that was used for the assessment of the tendon properties): Ap, aponeurosis; GM, m. gastrocnemius medialis; VL, m. vastus lateralis; F, fiber; M, myo-tendinous junction; Free, free tendon; nr, not reported. Sig (i.e., significance): *p < 0.05; +*p* < 0.01; -*p* > 0.05.

### Methodological quality and risk of bias assessment

The results of the methodological quality assessment of the included studies showed a range of achieved scores from 61% to 99% with a mean and standard deviation of 71% ± 9% (Table 3), indicating appropriate methodological qualities for most studies. Fourteen of the 27 included studies investigated mechanical, material, as well as morphological properties (i.e., stiffness, Young’s modulus, and CSA), which is essential in order to clarify if a change in tendon stiffness was based on alterations of the material properties and/or tendon hypertrophy.

**Table 3:** **Methodological quality and risk of bias assessment of the included studies**

**Study**	**Methodological quality**																																		**Risk of bias**					
	**Internal validity**																**Statistical validity**			**External validity**														**Total score [%]**	**Sequence**	**Allocation**	**Blinding**	**Outcome**	**Report**	**Other**
	**1.1** ^**a**^	**1.2** ^**a**^	**1.3** ^**a**^	**1.4** ^**a**^	**2.1A** ^**b**^	**2.1B** ^**b**^	**2.1C** ^**b**^	**2.1D** ^**b**^	**2.1E** ^**b**^	**2.1F** ^**b**^	**2.1G** ^**b**^	**2.2A** ^**b**^	**2.2B** ^**b**^	**3A** ^**b**^	**3B** ^**b**^	**Score [%]**	**4** ^**a**^	**5** ^**a**^	**Score [%]**	**6.1** ^**a**^	**6.2** ^**a**^	**7A** ^**b**^	**7B** ^**b**^	**7C** ^**b**^	**7D** ^**b**^	**7E** ^**b**^	**7F** ^**b**^	**8A** ^**b**^	**8B** ^**b**^	**8C** ^**b**^	**8D** ^**b**^	**8E** ^**b**^	**Score [%]**							
Albracht et al., 2013 [15]	+	-	-	+	-	+	+	+	-	+	-	/	/	-	+	51	+	-	50	+	+	+	+	+	+	+	+	-	+	+	+	+	95	65	Unclear	Unclear	Unclear	Yes	Yes	Yes
Arampatzis et al., 2007 [29]	+	+	+	+	-	+	+	+	-	+	-	+	+	+	-	87	+	-	50	+	+	+	+	+	+	+	+	+	+	+	+	-	95	77	Unclear	Unclear	Unclear	Yes	Yes	Yes
Arampatzis et al., 2010 [28]	+	+	+	-	-	+	+	+	-	+	-	+	+	+	-	72	+	-	50	+	+	+	+	+	+	+	+	+	+	+	+	-	95	72	Unclear’	Unclear’	Unclear	Yes	Yes	Yes
Bohm et al. [31]	+	+	+	+	-	+	+	+	+	+	+	+	+	+	+	98	+	+	100	+	+	+	+	+	+	+	+	+	+	+	+	+	100	99	Unclear	Unclear	Unclear	Yes	Yes	Yes
Carroll et al., 2011 [61]	+	+	+	-	+	-	-	-	-	+	+	+	+	-	+	70	+	-	50	+	+	+	-	+	+	+	+	+	+	+	+	+	96	72	Unclear’	Unclear’	Yes	Yes	Yes	Yes
Fletcher et al., 2010 [17]	+	-	-	+	-	+	+	-	+	+	-	/	/	+	+	60	+	-	50	+	+	+	+	+	+	+	+	+	+	+	+	+	100	70	Unclear	Unclear	Unclear	Yes	Yes	Yes
Fouré et al., 2009 [71]	+	-	-	+	-	+	-	-	-	-	-	/	/	+	+	46	+	-	50	+	+	-	-	+	-	+	+	+	+	+	+	+	88	61	Unclear	Unclear	Unclear	Unclear	Yes	Yes
Foure et al., 2010a,b, 2011 [38,56,57]	+	-	+	+	-	+	-	-	-	+	-	-	+	+	+	63	+	-	50	+	+	-	-	+	-	+	+	+	+	+	+	+	88	67	Unclear	Unclear	Unclear	Yes	Yes	Yes
Foure et al., 2013 [43]	+	-	+	+	-	+	-	-	-	+	-	-	+	+	+	63	+	-	50	+	+	-	-	+	-	+	+	+	+	+	+	+	88	67	Unclear	Unclear	Unclear	Yes	Yes	Yes
Hansen et al., 2003 [62]	+	-	+	-	-	-	-	+	+	+	-	+	+	-	+	56	+	-	50	+	+	-	+	+	+	+	+	+	+	+	+	+	96	67	Unclear’	Unclear’	Unclear	Yes	Yes	Yes
Hougthon et al., 2013 [39]	+	+	+	+	-	+	+	+	+	+	-	-	+	+	+	84	+	+	100	+	+	-	-	+	+	+	+	+	+	+	+	+	92	92	Unclear	Unclear	Unclear	Yes	Yes	Unclear^+^
Kongsgaard et al., 2007 [24]	+	+	+	-	+	+	-	+	-	+	-	+	+	+	+	74	+	-	50	+	+	+	-	+	+	+	+	+	+	+	+	+	96	73	Unclear’	Unclear’	Yes	Yes	Yes	Yes
Kubo et al., 2001a,b [37,42]	+	+	+	-	-	-	-	-	-	-	+	+	-	+	+	66	+	-	50	+	+	+	+	+	+	+	+	+	+	+	+	+	100	72	Unclear’	Unclear’	Unclear	Yes	Yes	Yes
Kubo et al., 2002 [25]	+	-	+	-	-	-	-	-	-	-	+	+	-	+	+	52	+	-	50	+	+	+	-	+	+	+	+	+	+	+	+	+	96	66	Unclear’	Unclear’	Unclear	Yes	Yes	Yes
Kubo et al., 2003 [63]	+	+	+	+	-	-	-	-	-	-	+	/	/	+	+	86	+	-	50	+	+	+	-	+	+	+	+	+	+	+	+	+	96	77	Unclear	Unclear	Unclear	Yes	Yes	Yes
Kubo et al., 2006a [40]	+	-	+	-	-	-	-	+	-	+	+	+	-	+	-	49	+	-	50	+	+	+	+	+	+	+	+	+	+	+	+	-	95	65	Unclear’	Unclear’	Unclear	Yes	Yes	Yes
Kubo et al., 2006b [87]	+	-	+	+	+	-	-	+	-	+	+	+	+	+	+	80	+	-	50	+	+	+	+	+	+	+	+	+	+	+	+	+	100	77	Unclear	Unclear	Unclear	Yes	Yes	Yes
Kubo et al., 2006c [88]	+	-	+	-	+	-	-	+	-	+	-	+	+	+	-	56	+	-	50	+	+	+	+	+	+	+	+	+	+	+	+	-	95	67	Unclear’	Unclear’	Unclear	Yes	Yes	Yes
Kubo et al., 2007 [59]	+	-	+	-	-	-	-	+	-	+	+	+	-	+	+	56	+	-	50	+	+	+	+	+	+	+	+	+	+	+	+	+	100	69	Unclear’	Unclear’	Unclear	Yes	Yes	Yes
Kubo et al., 2009 [60]	+	-	+	-	+	-	-	+	-	+	-	+	+	+	-	56	+	-	50	+	+	+	+	+	+	+	+	+	+	+	+	-	95	67	Unclear’	Unclear’	Unclear	Yes	Yes	Yes
Kubo et al., 2010 [52]	+	-	+	+	-	-	-	+	-	+	+	+	+	+	+	78	+	-	50	+	+	+	+	+	+	+	+	+	+	+	+	+	100	76	Unclear	Unclear	Unclear	Yes	Yes	Yes
Kubo et al., 2012 [72]	+	-	+	+	-	-	-	+	-	+	+	+	+	+	-	70	+	-	50	+	+	+	+	+	+	+	+	+	+	+	+	+	100	73	Unclear	Unclear	Unclear	Yes	Yes	Yes
Malliaras et al., 2013 [41]	+	+	+	+	+	-	-	+	-	+	+	-	-	+	+	80	+	-	50	+	+	+	+	+	+	+	+	+	+	+	+	+	100	77	Unclear	Yes	Yes	Yes	Yes	Yes
Seynnes et al., 2009 [30]	+	+	+	-	+	-	-	+	+	-	-	+	+	+	+	78	+	-	50	+	+	+	-	+	+	+	+	+	+	+	+	+	96	74	Unclear’	Unclear’	Unclear	Yes	Yes	Yes

Methodological quality: 1 Study design (1.1 Mechanical properties, 1.2 Material properties, 1.3 Morphological properties, 1.4 Control group), 2 Methods (2.1 Mechanical properties, 2.1A Object of investigation, 2.1B Gravitational forces, 2.1C Axes misalignment, 2.1D Antagonistic muscle activation, 2.1E Lever arm measured, 2.1F Joint angle change, 2.1G Used multiple trials, 2.2 Morphological properties, 2.2A MRI, 2.2B different positions), 3 Cofactors (3A Gender, 3B Activity level), 4 Statistical tests, 5 Power analysis, 6 Eligibility (6.1 Participants, 6.2 Variables), 7 Description exercise protocol (7A Intensity, 7B Duration single stimulus, 7C Repetitions, 7D Sets, 7E Weeks, 7F Times per week), 8 Description participants (8A Gender, 8B Age, 8C Body height, 8D Body weight, 8E Activity level). The single criteria were rated (+, point; -, no point; /, not included) and used to calculate the quality score for each category (i.e., internal, statistical, and external validity). The average of the three scores gives the total score. ^a^A full point was assigned to each sub-category for the calculation of the score in the respective validity section (assigned points/possible points * 100). ^b^The sub-categories of the respective block were pooled to a single point (assigned points/possible points). Risk of bias [46]: Sequence, adequate sequence generation; Allocation, allocation concealment; Blinding, blinding outcome assessor; Outcome, incomplete outcome data; Report, selective outcome reporting; Other, other sources of bias. Judgments: Yes, low risk of bias; Unclear, insufficient information reported (’, only one group; ^+^, significant difference of baseline tendon cross-sectional area values between the control and training group). The three studies of Foure et al. [38,56,57] and the two studies of Kubo et al. [37,42] were merged as one, since the results of one intervention were reported in different publications.

**Figure 2:**
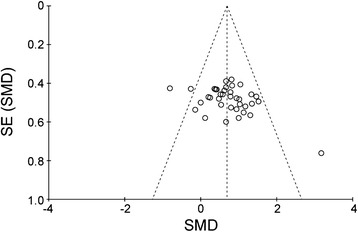
**Funnel plot of standardized mean differences (SMDs) against standard error of the mean (SE).** Values represent the tendon stiffness from all studies included in the meta-analysis.

The risk of bias assessment indicated a low risk of bias in three interventions [24,41,61]. The judgment for the other included studies was problematic, because the randomization process, concealment of allocation, and blinding of the assessor to the data were not reported and, therefore, unclear (Table 3). The funnel plot of tendon stiffness from all included studies appeared symmetrical and, thus, indicates low risk of publication bias (Figure 2).

### Meta-analysis of intervention effects

**Figure 3:**
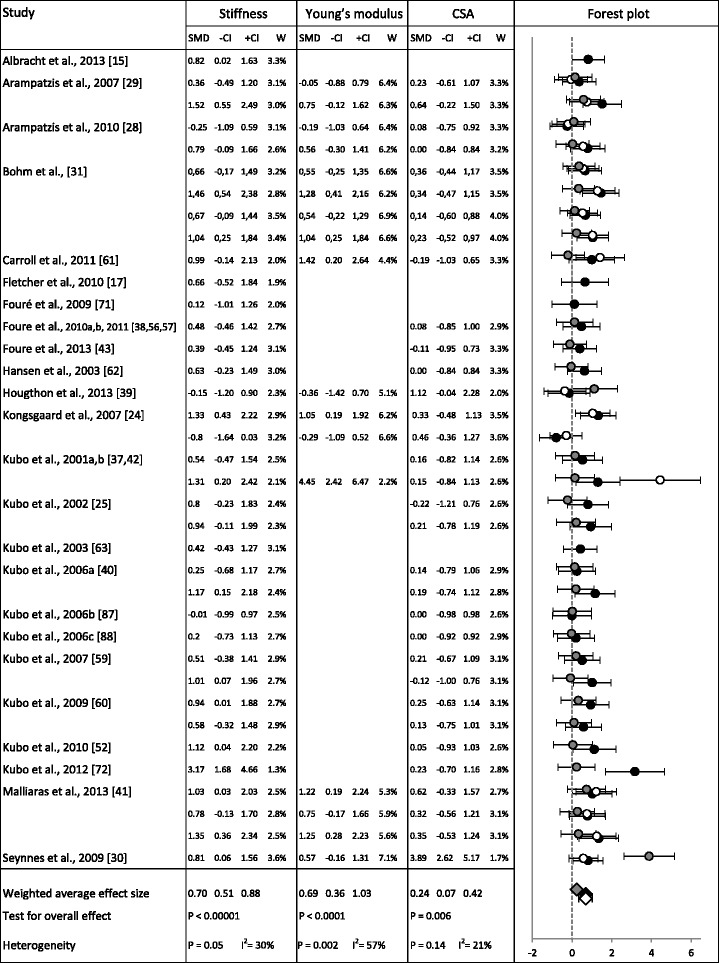
**Forest plot for the meta-analysis of the effect of mechanical loading on tendon properties.** Illustrated are the exercise intervention-induced changes on tendon stiffness (black), Young’s modulus (white), and cross-sectional area (CSA, gray), respectively, featuring the single-study effect sizes (SMD, circles), the corresponding confidence intervals (CIs, error bars), and study weight in the overall comparison (W) as well as the respective weighted average effect sizes (random-effects model, diamonds) with the overall effect test and heterogeneity analysis.

The weighted average effect size for the tendon stiffness was 0.70 (CI 0.51, 0.88), 0.69 (CI 0.36, 1.03) for tendon Young’s modulus and 0.24 (CI 0.07, 0.42) for tendon CSA, indicating greater intervention effects on stiffness and Young’s modulus compared to CSA (Figure 3). The overall intervention effect was significant for all three parameters (*p* < 0.05). Heterogeneity was significant for stiffness and Young’s modulus (*p* < 0.05), but not for CSA (*p* = 0.14), with a moderate heterogeneity of 30% and 21% for stiffness and CSA, respectively, and a substantial heterogeneity of 57% for Young’s modulus [50]. Figure 3 presents a forest plot, including the SMDs and corresponding CIs for tendon stiffness, Young’s modulus, and CSA of all included interventions as well as the respective weighted average effect sizes with the overall effect test and heterogeneity analysis results.

### Subgroup analysis

**Figure 4:**
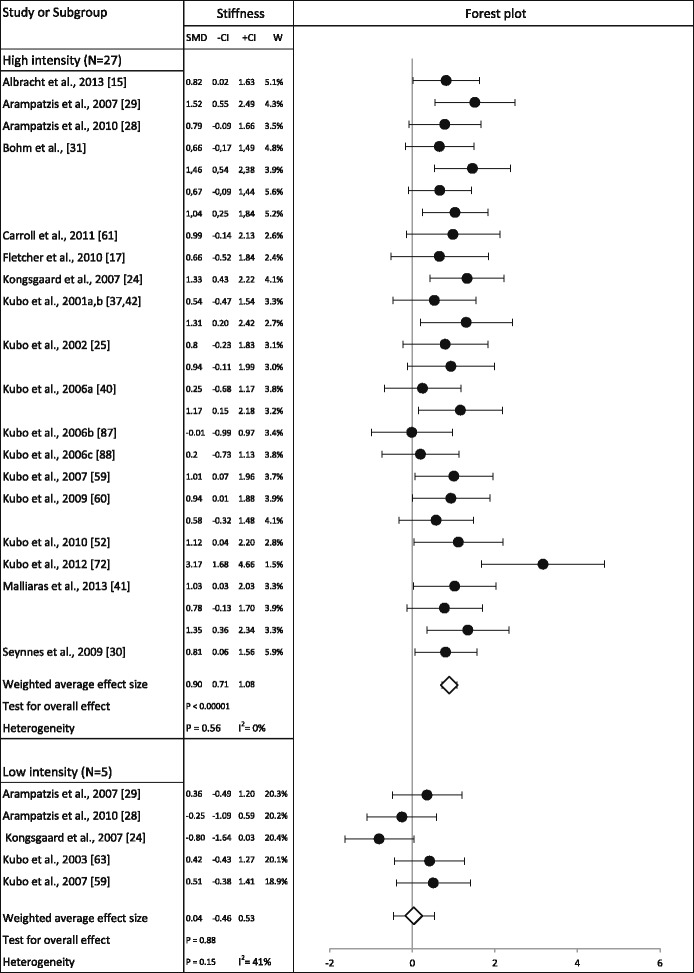
**Forest plot for the meta-analysis of the effect of mechanical loading on tendon properties.** Illustrated are the exercise intervention-induced changes on tendon stiffness (black), Young’s modulus (white), and cross-sectional area (CSA, gray), respectively, featuring the single-study effect sizes (SMD, circles), the corresponding confidence intervals (CIs, error bars), and study weight in the overall comparison (W) as well as the respective weighted average effect sizes (random-effects model, diamonds) with the overall effect test and heterogeneity analysis.

The subgroup analysis on the loading intensity showed that pooling interventions using muscle contraction intensities higher than 70% of MVC or RM (*N* = 27) and those using lower intensities (*N* = 5) resulted in a weighted averaged effect size of tendon stiffness of 0.90 (CI 0.71, 1.08) and 0.04 (CI −0.46, 0.53), respectively. The difference between the high and low intensity subgroup was statistically significant (*p* < 0.00001). No heterogeneity was found between the studies using high intensities (*p* = 0.56, *I*^2^ = 0%). The forest plot in Figure 4 contains the SMDs and corresponding CIs for tendon stiffness separated for interventions featuring high and low loading intensities as well as the respective weighted average effect sizes with the overall effect test and heterogeneity analysis.

When analyzing the interventions that used a high loading intensity in regard to the type of muscle contraction, the weighted averaged effect sizes of tendon stiffness showed no statistically significant (*p* > 0.5) difference between isometric (*N* = 15, SMD = 0.95, CI 0.66, 1.24), concentric-eccentric (*N* = 8, SMD = 0.82, CI 0.49, 1.15), and purely eccentric (*N* = 2, SMD = 1.04, CI 0.37, 1.72) contraction type.

The subgroup analysis of the intervention duration showed that the weighted average effect sizes of tendon stiffness were 0.91 for the interventions using longer durations (≥12 weeks: *N* = 23, CI 0.71, 1.12) and 0.81 for the shorter ones (8 to 12 weeks: *N* = 4, CI 0.33, 1.29). No statistical significant difference was found between the two durations (*p* = 0.7).

## Discussion

The present meta-analysis assessed the effect of chronic mechanical loading on the adaptive responses of tendon mechanical (stiffness), material (Young’s modulus), and morphological (CSA) properties reported in the recent literature. Twenty-seven studies, which provided an overall number of 37 separate exercise interventions (participants in total *N* = 264), were included in the analysis. The weighted averaged effect size of the intervention-induced adaptations was 0.70 for tendon stiffness (*N* = 37), 0.69 for Young’s modulus (*N* = 17), and 0.24 for CSA (*N* = 33), indicating a moderate to large effect for the first two parameters and a small to moderate effect for the latter. The overall intervention effect for stiffness, Young’s modulus, and CSA was significant, regardless of the variety of applied loading regimens. However, the significant heterogeneity of stiffness and Young’s modulus between the included interventions indicated that the different levels of the loading conditions might have affected the adaptive responses. The subgroup analysis revealed that high loading intensities are more effective compared to low intensities to induce adaptive responses whereas the type muscle contraction seems irrelevant. This meta-analysis gives further evidence for the plasticity of human tendon mechanical, material, and morphological properties *in vivo* in response to chronic loading of various types. Moreover, the analysis showed that the adaptive response of the tendon to intervention-induced chronic mechanical loading might be more pronounced for the material compared to morphological properties.

The averaged effect size of the intervention-based changes of tendon stiffness was 0.70, featuring a significant overall effect of all included exercise interventions. Out of the 37 interventions that measured tendon stiffness, 26 showed SMDs above 0.5 (i.e., medium to large effects [64]). Therefore, the present meta-analysis emphasizes the adaptive potential of tendons to increased mechanical loading, which was quite consistently shown despite the marked variety of loading protocols. However, the significant heterogeneity of tendon stiffness changes between studies indicated that especially the different levels of the applied loading conditions (e.g., intensity, duration of single loading cycle, repetitions, sets, intervention duration, and training frequency per week) and general exercise conditions (e.g., type of muscle contraction (isometric, concentric, or eccentric) applied repetitively or statically, differences in joint angles that affect the tendon lever arm length and, thus, acting stress on the tendon) may considerably affect tendon adaptive responses. For example, Arampatzis et al. [28,29], Kongsgaard et al. [24], and Malliaras et al. [41] investigated the effect of the magnitude of the mechanical load by means of low and high muscle contraction intensities. The studies reported a significant increase of tendon stiffness solely following the training using the high contraction intensities (i.e., 90% MVC, 70% RM, 80% eccentric RM, respectively). The conducted subgroup analysis confirmed the importance of high tendon loading intensities for tendon adaptation. Analyzing the interventions that used muscle contraction intensities higher than 70% of MVC or RM and those using lower intensities revealed significantly different weighted averaged effect sizes of tendon stiffness of 0.90 and 0.04, respectively. No heterogeneity (*I*^2^ = 0%) between studies using high intensities was found. Furthermore, considering the high contraction intensity studies, the analysis further indicates that the effect of the type of muscle contraction was only minor (Table 2). The subgroups of either isometric, concentric-eccentric, or purely eccentric contraction type showed comparable (statistically insignificant) weighted averaged effect sizes of 0.95, 0.82, and 1.04, respectively. Therefore, we can argue that the level of tendon loading (in terms of muscle contraction intensity) determines the effect on tendon adaptation independent of the muscle contraction type, which may explain the lack of differences between the interventions using different muscle contraction types. This assumption is in accordance to reports from earlier *in vitro* studies, suggesting that loading intensity-related tendon cell deformation is an important stimulus that affects catabolic and/or anabolic cellular and molecular adaptive responses [65,66]. With increasing strain, a loss of collagen crimp and an increase in fiber recruitment was observed [67,68], which very likely results in an increased number of cells being deformed [69] inducing adaptive processes in an intensity-dependent manner [65,66,70].

However, several of the included studies evidenced that besides the magnitude of tendon loading additional loading and exercise conditions may affect tendon adaptation, e.g., loading frequency [28], loading rate [31], joint angle [40], loading duration [31,37,42], and repetitive vs. static loading [31,60].

Furthermore, the effect of plyometric training on tendon properties seems yet ambiguous, since the five plyometric training interventions [31,38,56,57,59,71] included in the present meta-analysis reported controversial results. The changes in tendon stiffness ranged from +28% [31] to −9% [39]. However, only the 27% increase reported by Foure et al. [38,56,57] reached statistical significance. The different jumping exercises, uncontrolled [38,39,43,56,71] or comparably low (40% RM [59]) tendon load magnitude, and dissimilar intervention durations (8 to 14 weeks) might be the reason for the inhomogeneous findings. Comparing dynamic (concentric-eccentric) and isometric training with plyometric training, Kubo et al. [59] and Bohm et al. [31] reported a statistically significant increase of Achilles tendon stiffness solely following the dynamic and isometric but not after the plyometric training. Bohm et al. [31] suggested that the short loading duration during jumping constrains the transduction of the mechanical stimulus to the cellular level due to the viscosity of the tendinous tissue and, therefore, may not be optimal to facilitate adaptive responses of the tendon.

In regard to the duration of the exercise intervention, several of the included studies featuring a duration of 8 weeks found significant adaptations of tendon stiffness [17,25,30], indicating that tendons already respond to increased mechanical loading within 2 months. Pooling the interventions featuring a high intensity with respect to the intervention duration in the subgroup analysis, the weighted average effect sizes of tendon stiffness were 0.91 for the interventions (*N* = 23) using longer durations (≥12 weeks) and 0.81 (*N* = 4) for the shorter ones (8 to 12 weeks) without a statistically significant difference. The present analysis showed that shorter intervention durations (8 to 12 weeks) may induce tendon adaptive responses; however, longer durations (≥12 weeks) seem to be more efficient, and their effect has been clearly demonstrated in many studies. For example, Kubo et al. [72,73] showed that within the time-course of training, Achilles and patellar tendon stiffness did not increase significantly after 2 months but reached statistical significance at the end of the 3-month training period.

The present meta-analysis solely included data of Achilles and patellar tendons. However, as to be expected, similar loading protocols on different types of tendons induced similar adaptive responses [58]. Therefore, evidence-based interventions that facilitate tendon adaptation should be applicable to various tendons and prove valuable in regard to athletic training as well as the therapy and prevention of tendon injuries.

Increases in tendon stiffness may be a result of either change in tendon material properties (i.e., Young’s modulus) and/or tendon morphological properties (i.e., cross-sectional area and tendon rest length). Several studies reported increases in tendon CSA following training interventions [24,28-31]. However, it is feasible that no such reports exist for an exercise-induced change of tendon rest length, which hence can be excluded from being a relevant adaptive mechanism in response to increased mechanical loading. Regardless of the differences between the applied loading regimens, the averaged effect size for Young’s modulus (*N* = 17) was 0.69 and for CSA 0.24 (*N* = 33). The overall intervention effect was significant for both Young’s modulus and CSA, and the heterogeneity between studies was significant for Young’s modulus and moderate for CSA. As averaged effect size of stiffness and Young’s modulus were very similar and comparably higher as the CSA effect size, we can argue that the increase in stiffness may be primarily attributed to alterations of the material properties rather than morphological properties. Changes of the material properties were mentioned to be an early mechanism for increased stiffness, whereas tendon hypertrophy could be a long-term effect of mechanical loading [19,32]. Several studies included in the present meta-analysis found an increase in tendon Young’s modulus following the exercise interventions without changes in the tendon CSA [26,28,42], supporting the assumption that material properties demonstrate greater plasticity and change more instantaneous in response to enhanced chronic mechanical loading. Taking into account that the average duration of all included interventions was 12.9 ± 4.5 weeks (two studies with longer durations than 14 weeks: running training [62] and low load resistance bodyweight training [63]), the reason for the small averaged effect size of CSA in contrast to the larger effects of Young’s modulus may be the relatively short intervention durations. Yet, tendon hypertrophy could be more pronounced following longer periods of loading (i.e., habitual loading) compared to durations commonly used in exercise interventions.

Besides physiological adaptive responses of the tendon to increased mechanical loading in terms of a functional relevant improvement, excessive mechanical loading (i.e., overloading) was considered an important factor in the etiology of tendinopathy [20,34,35], which is characterized by activity-related pain, focal tendon tenderness, and decreased strength and flexibility [32]. It was suggested that repetitive strains, though below the failure threshold of the tendon, cause tendon micro-injuries and subsequently tendon inflammation, which may contribute to the development of tendon degeneration [22]. None of the included interventions reported a significant dropout of participants due to clinical symptoms of overloading. Nevertheless, adequate regeneration times and slower adaptation rates of tendon compared to muscle [72-74] should be considered in an exercise intervention to avoid episodes of high tendon strain and stress [75] that may cause maladaptation and tendon damage.

The appropriate investigation of tendon properties needs to include numerous methodological considerations. The total methodological quality score used in the present meta-analysis ranged from 61% to 99% with a mean of 71% ± 9%, indicating adequate to high methodological qualities for most studies and, thus, study validity. However, several aspects of the internal study validity (i.e., study design, methods, and co-factors) were not considered in every study. First, only 17 of the 37 included interventions reported the values of stiffness, Young’s modulus, and CSA and, therewith, provided a complete examination of the adaptive processes of the mechanical, material, and morphological tendon properties and their interaction. Only about half (i.e., 19) of the interventions included a control group. To determine the tendon CSA, four studies (six interventions) used ultrasonographic images instead of MRI [76] for the manual segmentation, although the reliability for this method was reported to be poor [45]. However, advancements in the analysis of ultrasound signals (e.g., ultrasound tissue characterization) for the assessment of tendon dimensions are promising [77,78] and may be attractive for the detection of intervention-induced increases of tendon CSA in future studies. With regard to the measurement and calculation of the tendon force, tendon elongation, and CSA, not a single study considered all relevant methodological aspects (e.g., accounting for gravitational forces, axes misalignment of joint and dynamometer, averaging multiple trials to reliably assess tendon elongation, measuring the tendon moment arm directly), which affects the validity of the applied method. In consequence, the score for the internal validity was in average only 66% ± 15% (range: 49% to 98%). Considering the statistical validity, all studies applied appropriate statistical tests, but only two studies [31,39] calculated the effect size to estimate the effect of the intervention-induced tendon adaptations. Furthermore, there was a clear deficit in controlling and reporting all relevant loading conditions (e.g., intensity, duration of single loading cycle) [38,39,43,56,71], compromising the comparability of the results between interventions and their interpretations in regard to potential causalities. Nevertheless, a mean external validity score of 96 ± 4% (range 88% to 100%) indicated a high external validity of all included studies. Although already considered in most of the included studies, future investigations on tendon adaptation should account for these methodological quality criteria to ensure high study validity. The risk of bias assessment was difficult, since important information were not reported in most articles. In particular, details of the randomization process, concealment of allocation, and/or blinding of the assessor to the outcome data were missing in 34 of the 37 interventions, and therefore, the risk of bias judgment was inadequate for most included interventions. Only three studies [24,41,61] provided the necessary information, and the assessment indicated a low risk of bias. However, the judgment of the other domains (i.e., incomplete outcome data, selective outcome reporting, and other sources of bias) indicated a low risk of bias for almost every included study. Future investigations should account for an appropriate consideration and/or presentation of these aspects to allow for risk of bias estimation. Although the risk of bias assessment could not be performed adequately due to a lack of information, the overall assessment together with the methodological quality scale suggests an appropriate validity of the included studies. Furthermore, the funnel plot indicated a low risk of publication bias. Therefore, the outcome of the present meta-analysis provides profound evidence.

The current review and meta-analysis may feature some limitations in regard to the sample sizes, recruited participants, and durations of the included interventions. All included studies were performed on small sample sizes (6 to 15 participants), most likely due to the great study effort, and thus, conclusions with regard to a greater population based on solely one intervention should be drawn carefully. However, the present meta-analysis on recent literature confirmed the effects of chronic loading on the adaptation of mechanical, material, and morphological tendon properties. To a greater part, the included participants were male (237 of 264) and involved in recreational activity (approximately 164 of 264), which could have biased the generalizability of the study outcomes to a greater mixed-gender population with a different activity profile. Furthermore, the duration of 35 of the 37 included interventions was short term (≤14 weeks). However, longer durations may affect the adaptive responses of the separate tendon properties (material and morphological) in a different way. Moreover, the present meta-analysis only considered studies in the English language.

## Conclusions

In conclusion, the present meta-analysis on the effect of chronic mechanical loading on human tendon adaptation *in vivo* included 27 studies featuring 37 separate exercise interventions. The meta-analysis showed that tendons are highly responsive to increased mechanical loading and adapt through changes of their mechanical, material, and morphological properties. Intervention-induced changes in tendon stiffness seem to be more attributed to adaptations of the material rather than morphological properties. Based on the results of the present meta-analysis, we can conclude that high magnitude loading (i.e., muscle contraction intensity) is most effective to elicit tendon adaptation and that longer intervention durations (>12 weeks) are beneficial compared to shorter ones. The effect of muscle contraction type (isometric, concentric-eccentric, or isometric) seems insignificant; however, the review suggests that plyometric training may not be optimal to facilitate tendon adaptation.
